# Dataset on acido-biocatalysis of agro solid wastes in acidic medium for the conversion of pink solo *Carica papaya* seed oil to biodiesel fuel

**DOI:** 10.1016/j.dib.2024.110219

**Published:** 2024-02-21

**Authors:** O. Nyorere, S.I. Oluka, S.E. Onoji, R. Nwadiolu, T.F. Adepoju

**Affiliations:** a Agricultural Engineering Department, Delta State University of Science and Technology, Ozoro, Delta State, Nigeria; b Agricultural and Bioresource Engineering Department, Enugu State University of Science and Technology, Ozoro, Enugu State, Nigeria; c Petroleum and Natural Gas Processing Department, Petroleum Training Institute, Effurum, Delta State, Nigeria; d Department of Agricultural Economics, Faculty Agriculture, Delta State University of Science and Technology, Ozoro, Delta State, Nigeria; e Chemical Engineering Department, Delta State University of Science and Technology, Ozoro, Delta State, Nigeria

**Keywords:** Biofuel, Heteroatoms, Continuous extraction, Optimization, TGA, Zeta analysis, SEM-EDX

## Abstract

In an attempt to find a single stage conversion of high free fatty acid (FFA) of Pink Solo *Carica papaya* oilseed rather than the double steps, acidic catalyst was derived from burnt fermented sweet corn stock powder immersed in acidic environment, and was used to convert Pink Solo *Carica papaya* oilseed to biodiesel. The derived catalyst was characterized using TGA, ZETA, FTIR, SEM-EDX, XRF-FS, and BET analysis. Process modeling and optimization was carried out using response surface methodology (RSM) and artificial neural network (ANN). The produced biodiesel was quantified by determining its physicochemical parameters, and the strength of acidified catalyst (AC) was tested in reusability cycles. Dataset show the extracted oil is acidic (acid value >3.0 mg KOH/g oil). The produced AC showed the presence of Quartz (68%), Orthoclase (7.1%), ibise (9.8%), and illite (15%). Process modeling and optimization validated the optimum biodiesel yield of 99.02% (wt./wt.) at *X*_1_ = 78.42 (min), *X*_2_ = 2.19 (%wt.), and *X*_3_ = 5.969 for RSM_BBD_, and 99.97% (wt./wt.) at *X*_1_ = 70.41 (min), *X*_2_ = 5.40 (%wt.), and *X*_3_ = 6.00 for ANN_FA_. Catalyst recyclability test data undergoes 10 recycles, and the produced biodiesel qualities were in line with the recommended standard. The study concluded that the burnt sweet corn stock powder, when immersed in acid, can convert high FFA oil of Pink Solo *Carica papaya* to biodiesel in a single stage.

Specifications Table

**Table:** 

Subject	Energy
Specific subject area	Continuous Extraction, Fermentation, Thermal Treatment, Acid immersion, Characterization, Transesterification, Catalyst Reusability
Data format	Raw, Characterized, Analyzed, Modeled, Optimized, Recycled, Reused
Type of data	Table, Figure
Data collection	Oil was obtained from Pink Solo *Carica papaya* Seed The nature of oil was ascertain by determined the properties of oil via AOAC, 1997 procedural methods Acidified catalyst was obtained using fermented sweet corn stock powder immersed in an acid The nature of catalyst was ascertain by characterized with TGA, FTIR, ZETA, XRD-EDX, SEM, XRF-FS, and BET analysers Biodiesel was produced in a single stage process rather than the double steps Modeling and optimization was carried out via Response Surface Methodology and Artificial Neural Network The strength of the derived catalyst was tested by recyclability and reusability test
Data source location	Department of Agricultural and Bioresource Engineering, Enugu State University of Science and Technology [Longitude: 6.459964, Latitude: 7.548949], Enugu State, Nigeria.
Data accessibility	Repository name: Dataset on Acido-biocatalysis of Agro Solid Wastes in Acidic Medium for the Conversion of Pink Solo Carica papaya Seed oil to Biodiesel Fuel. DOI: 10.17632/ppzcrfccvb.1 Direct URL to data: https://data.mendeley.com/datasets/ppzcrfccvb/1

## 1 Value of the Data

• Datasets on the properties of Pink Solo *Carica papaya* seed oil reflected unsaturated oil.
• Catalyst analysis data proved the acidified catalyst was strong enough to convert the oil to biodiesel in single stage reaction.
• The produced biodiesel shows data value similar to that of recommended biodiesel standard (ASTM D6751 & EN 14214).
• Data on process modeling and optimization via RSM & ANN tools indicated GA predicted the optimum data values than BBD.
• The research established data on a variety of *Carica papaya* seed and corn stock

## 2 Background

Oil has been reportedly obtained from *Carica papaya* seed, but no single reports ever identify the variety of the *C. papaya* seed employed. Also, corn stock have been reportedly used to synthesized heterogeneous catalyst for biodiesel conversion, but literatures on the type of corn stock used have not been reported. Lastly, the conversion of high free fatty acid oil of *C. papaya* has been reportedly to required two steps conversion. These steps lead to high cost of biodiesel production as well as time wastage. Therefore, this study employed acidified corn stock powder of sweet corn for the conversion of pink solo *C. papaya* oil to biodiesel in a single step conversion. The acidified catalyst was characterized using FTIR, XRD-EDX, BET, TGA, ZETA, BET and XRF-FS analyser. The produced biodiesel was quantified by determined the properties with a view to determine its ability to replace conventional diesel.

Pink solo *Carica papaya* is a variety of papaya that grows up to 8 inches with a minimum temperature of 60 °C. The grown period required full sun and can be found surplus in spring period. It replication can occurs both sexually and asexually. It is propagated by seeds; but, vegetative propagation has been often used with the use of vitro and ex-vitro culture techniques. The pink solo *Carica papaya* seed is usually sampled by removing the seed from the edible fruits.

## 3 Data Description

Based on its attribute such as simple recovery, non polar nature, low latent heat of vaporization, and high selectivity, *n*-hexane was used as a solvent to obtained oil from the pink solo C. papaya seed. Acidified catalyst used for the conversion of high FFA oil to biodiesel was obtained from sweet corn stock powder. Process modeling and optimization were carried out in a single step. The data in this study include the properties of oil, experimental, predicted, thermal treatment, and characterization, analysis, calculated, and formulated. These datasets were structured into tables and figures to illustrate the conversion of high FFA pink solo C. papaya oil (PSCPO) to biodiesel in a single step batch reaction using acidified fermented sweet corn stock powder (AFSCSP). Table 1 depicts the experimental design variables for oil extraction. Three variables; *C. papaya* seed weight (CPSW), Extraction time (ET), and volume of solvent (VS) were considered for the optimum oil yield data.

**Table 1:** Variable factors levels for oil extraction design by RSM.

**Fig. 1:**
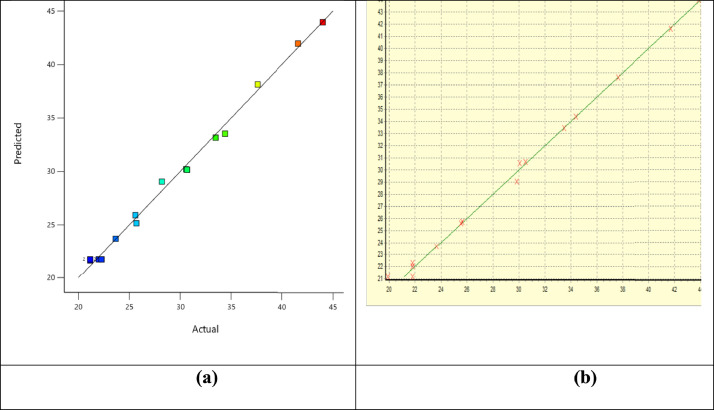
(a): Experimental vs. RSM-BBD Predicted values (b): Experimental vs. ANN-GA Predicted values.

The experimental and the predicted values datasets for both Box Behnken Design (BBD) and Genetic Algorithms (GA) are represented by the plots in Fig. 1(a, b). The analysis of variance (ANOVA) data that provide data for variables level of significant, probability level, the *F*-values, and the Fit statistics are displayed in Table 2a. The modeling polynomial quadratic equation that explained the variables interaction on the response oil yield in actual form is presented in Eq. (1).

**Table 2:** Analysis of variance for regression analysis.

Source	Sum of squares	df	Mean square	*F*-value		*p*-value	Level of significance
**Model**	881.03	9	97.89	229.00		<0.0001	significant
A	295.37	1	295.37	690.95		<0.0001	
B	0.7813	1	0.7813	1.83		0.2185	
C	130.98	1	130.98	306.40		<0.000	
AB	19.67	1	19.67	46.01		0.0003	
AC	4.41	1	4.41	10.32		0.0148	
BC	13.07	1	13.07	30.57		0.0009	
A²	309.39	1	309.39	723.75		<0.0001	
B²	52.16	1	52.16	122.01		<0.0001	
C²	24.90	1	24.90	58.26		0.0001	
Residual	2.99	7	0.4275	–		–	
Lack of fit	1.72	3	0.5732	1.80		0.2864	not significant
Pure error	1.27	4	0.3182	–		–	
Cor. total	884.02	16	–	–		–	

**RSM**				**ANN**			

*R*^2^	99.66%	–	–	*R*^2^	99.93%		
Adj. *R*^2^	99.23%	–	–	Adj. *R*^2^	99.86%		
Pred. *R*^2^	96.66%	–	–	Pred. *R*^2^	96.95%		
Std. Dev.	0.6538	–	–	Std. Dev.	2.594		

Transfer model equation in terms of coded value (−1 to +1):

(1) $$\begin{array}{ccc} \text{OY}\%(\text{wt}./\text{wt}.)\; & = & 21.78-6.08A-0.31B-4.05C-2.22AB+1.05AC\\ & & -\;1.81\text{BC}+8.57A_{1}^{2}+3.52B_{2}^{2}+2.43C_{3}^{2} \end{array}$$

**Fig. 2:**
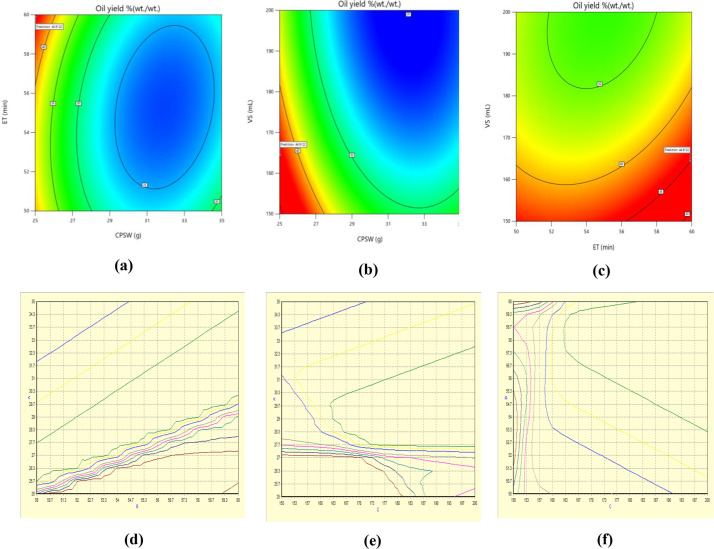
(a–c): RSM-BBD contour plots (d–f): ANN-GA contour plots.

The mutual relationship between the interactive factors (AB, AC, and BC) on the response (oil yield) was established and presented inform of the contour plots (Fig. 2[a–c] for RSM & 2[d–f] for ANN). The optimum predicted response by RSM and ANN were validated in triplicate, and an average values of 43.20% (wt./wt.) [*A* = 25.02 (wt.), *B* = 59.91 min, and *C* = 164.47 mL] and 44. 92% (wt./wt.) [*A* = 24.6 (wt.), *B* = 56.70 min, and *C* = 150.56 mL] were established. ANN shows supremacy over the RSM optimum validated yield. Presented in Table 3 are the datasets on the properties as well as the fatty acid compositions of the oil.

**Table 3:** Properties of extracted pink solo *C. papaya* oil.

Properties	Pink solo *C. papaya* oil
Moisture content (%)	<0.001
Specific gravity	0.945
Viscosity @ 25 °C/ (mm^2^/s)	12.24
Acid value (mg KOH/g oil)	4.90
%FFA (as oleic)	2.45
Peroxide (meq O_2_/kg oil)	10.56
Saponification value (mg KOH/g oil)	192.10
Iodine value (g I_2_/100 g oil)	77.92
Cetane number	57.18
Higher heating value	40.39
Mean molecular mass	29.15
Fatty acid compositions	
Palmitic acid (C16:0)	18.60
Oleic acid (C18:1)	70.43
Total unsaturation	89.03
Others (saturation)	10.97

**Fig. 3:**
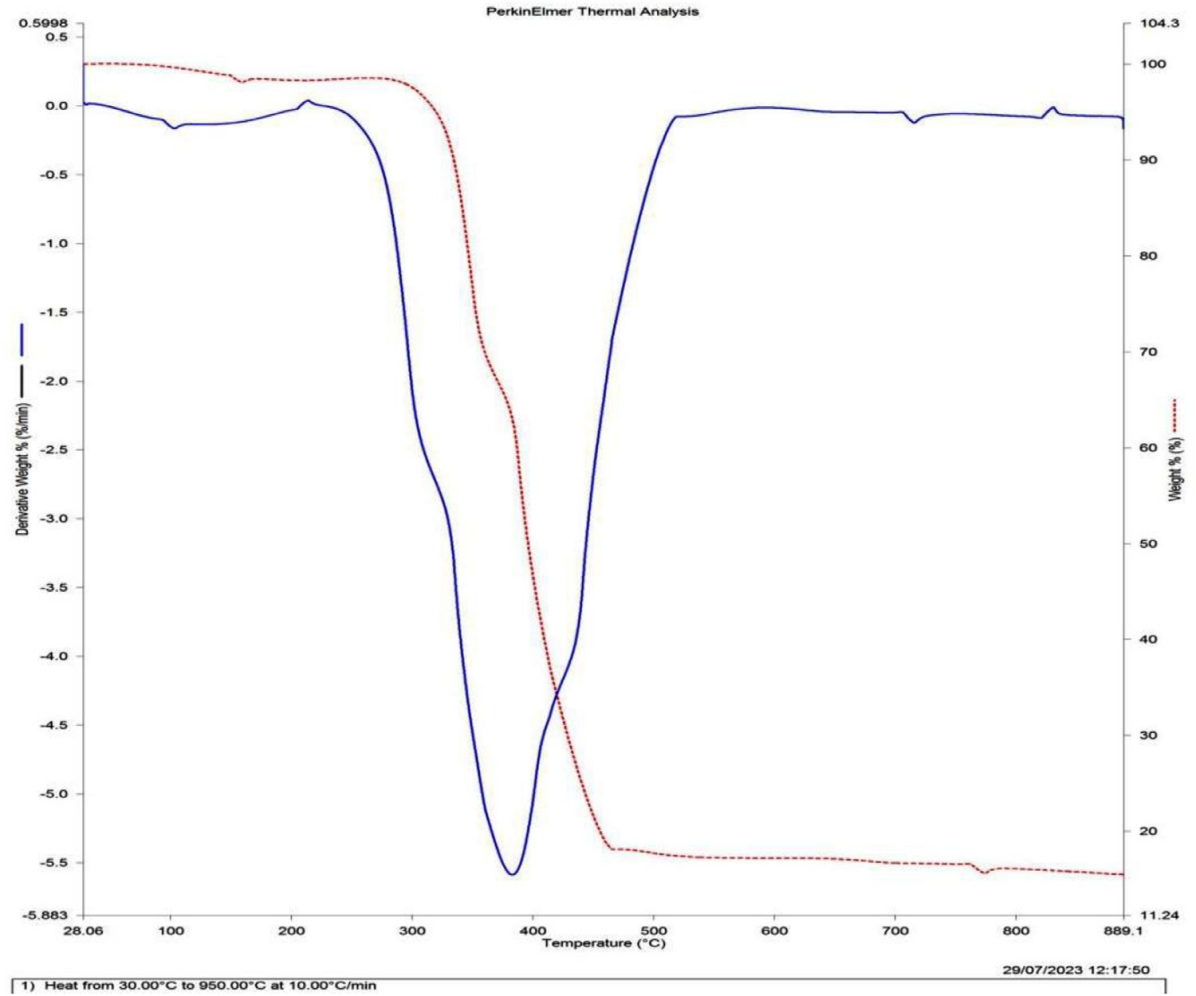
The TGA plots.

**Fig. 4:**
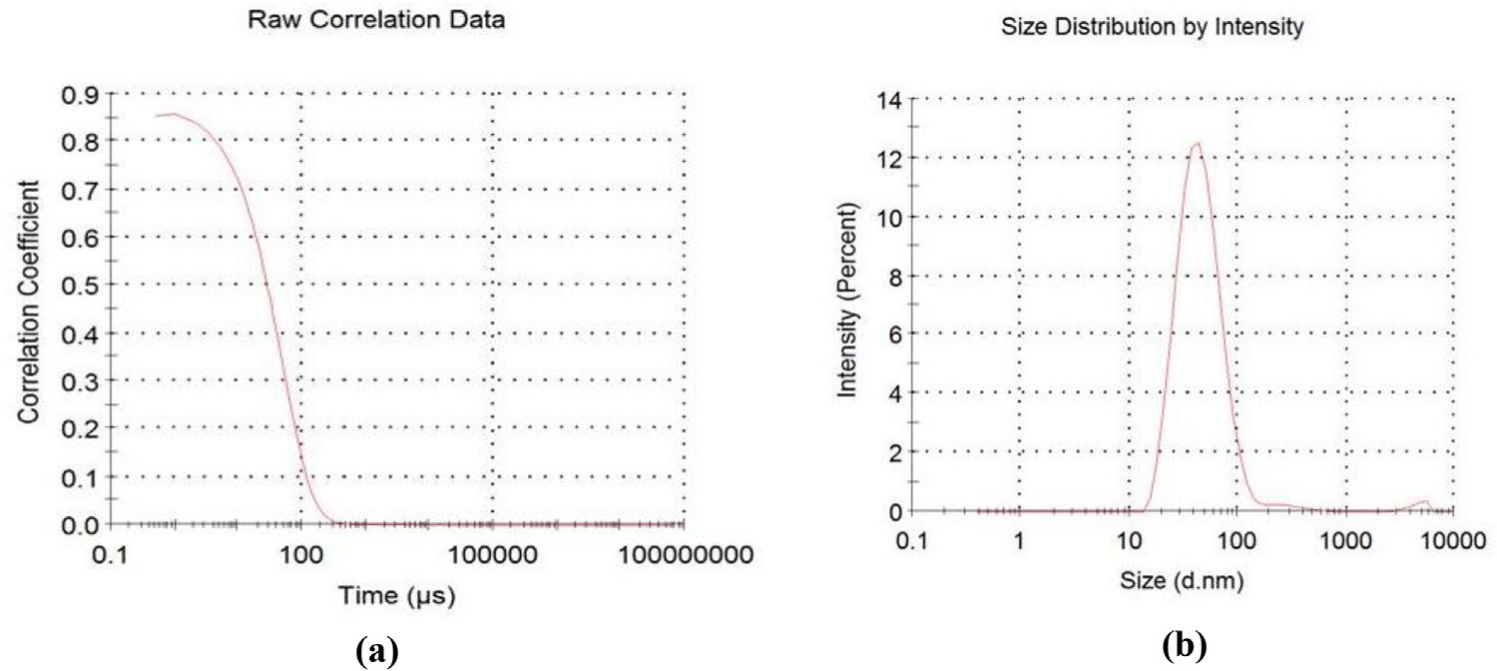
(a, b): ZETA plots.

**Fig. 5:**
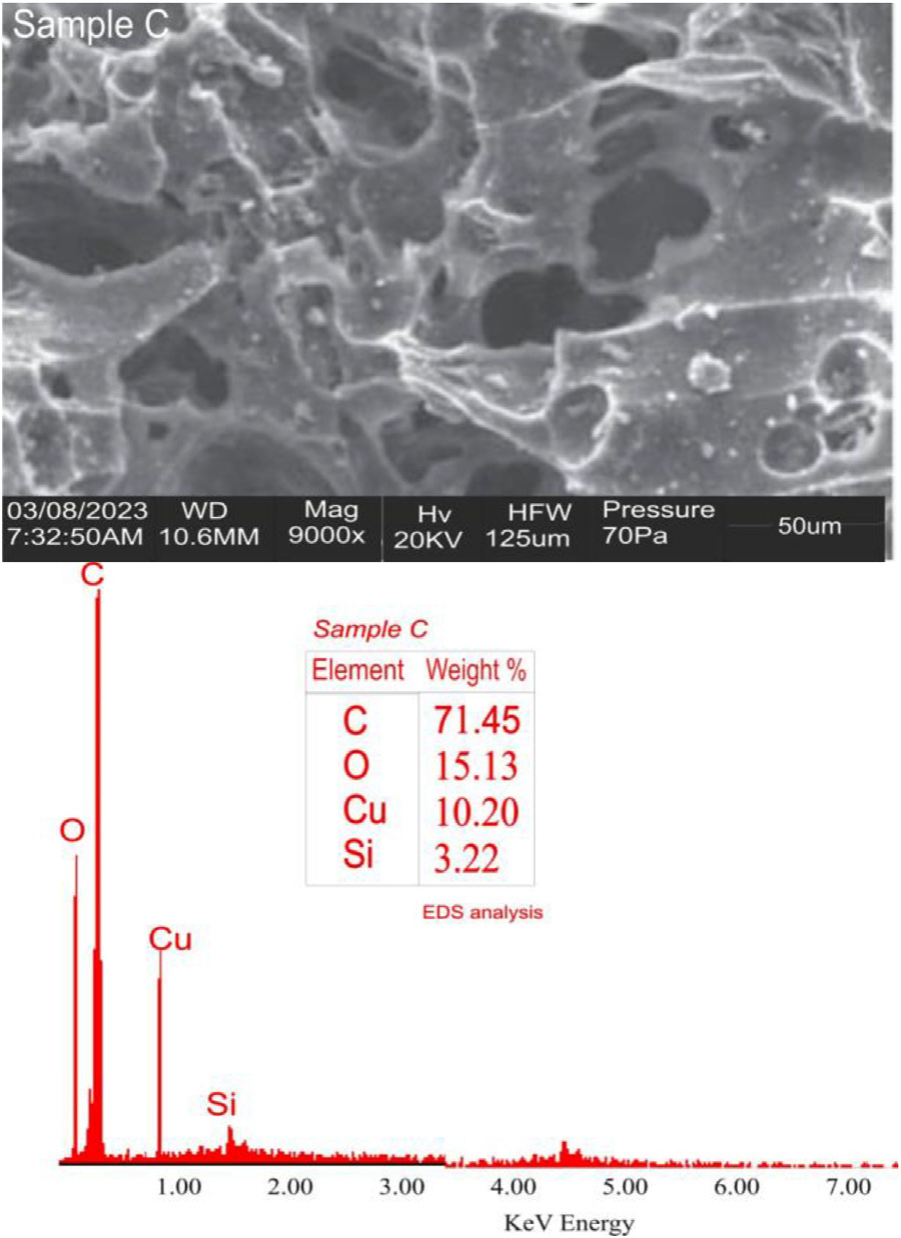
The SEM-EDS image.

**Fig. 6:**
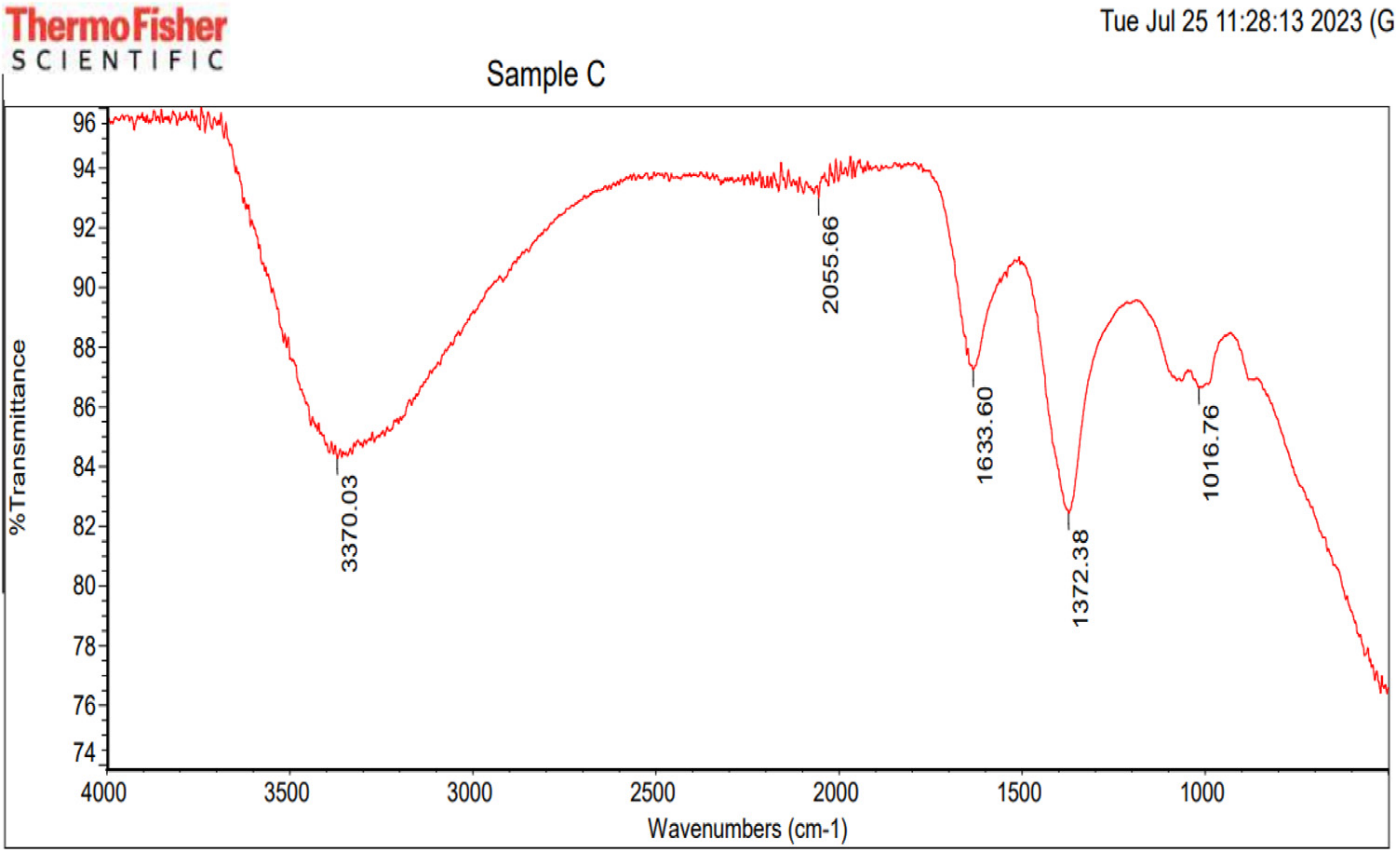
The FTIR image.

Datasets on the thermal treatment of the AFSCSP via TGA are showed in Fig. 3, while Fig. 4(a, b) displayed the datasets on the size distributions against intensity, as well as correlation coefficient against time as obtained by ZETA plots. Table 4 however explained the data on elemental analysis of the AFSCSP via XRF-FS, while BET analysis data value explaining the pore spacing, volume, and the surface area of the AFSCSP are indicated in Table 5. Displayed in Fig. 5 is the SEM-EDS image of the produced AFSCSP with morphological structure at magnification of 9000×. The FTIR analysis of the catalyst with respect to the functional group is presented in Fig. 6.

**Table 4:** Elemental analysis via XRF-FS.

Components	SiO_2_	Fe_2_O_3_	P_2_O_5_	SO_3_	CaO	MgO	K_2_O	Cl	Others
Concentration (%wt.)	10.986	2.114	4.298	2.537	13.095	3.689	36.301	16.412	10.5568
Mole (%)	11.895	0.861	1.970	2.061	15.452	5.954	26.452	30.116	5.239

**Table 5:** BET analysis showing the pore, volume, and the surface area.

Pore width	Cumulative pore volume	Cumulative surface area	dV(d)	dS(d)
1.7656	6.84E−02	1.23E+02	8.23E−02	9.32E+01
1.8469	7.39E−02	1.29E+02	6.73E−02	7.28E+01
1.9319	7.99E−02	1.35E+02	7.08E−02	7.33E+01
2.0208	8.47E−02	1.39E+02	5.37E−02	5.32E+01
2.1138	8.49E−02	1.40E+02	3.10E−03	2.93E+00
2.2111	9.11E−02	1.45E+02	6.35E−02	5.74E+01
2.3129	1.03E−01	1.55E+02	1.13E−01	9.81E+01
2.4194	1.13E−01	1.64E+02	9.47E−02	7.83E+01
2.5307	1.25E−01	1.74E+02	1.13E−01	8.96E+01
2.6472	1.41E−01	1.85E+02	1.31E−01	9.90E+01
2.7691	1.53E−01	1.94E+02	1.02E−01	7.40E+01

For biodiesel synthesis, the process modeling variables data considered for experimental design are displayed in Table 6.

**Table 6:** Variable factors levels data for biodiesel design by RSM.

**Fig. 7:**
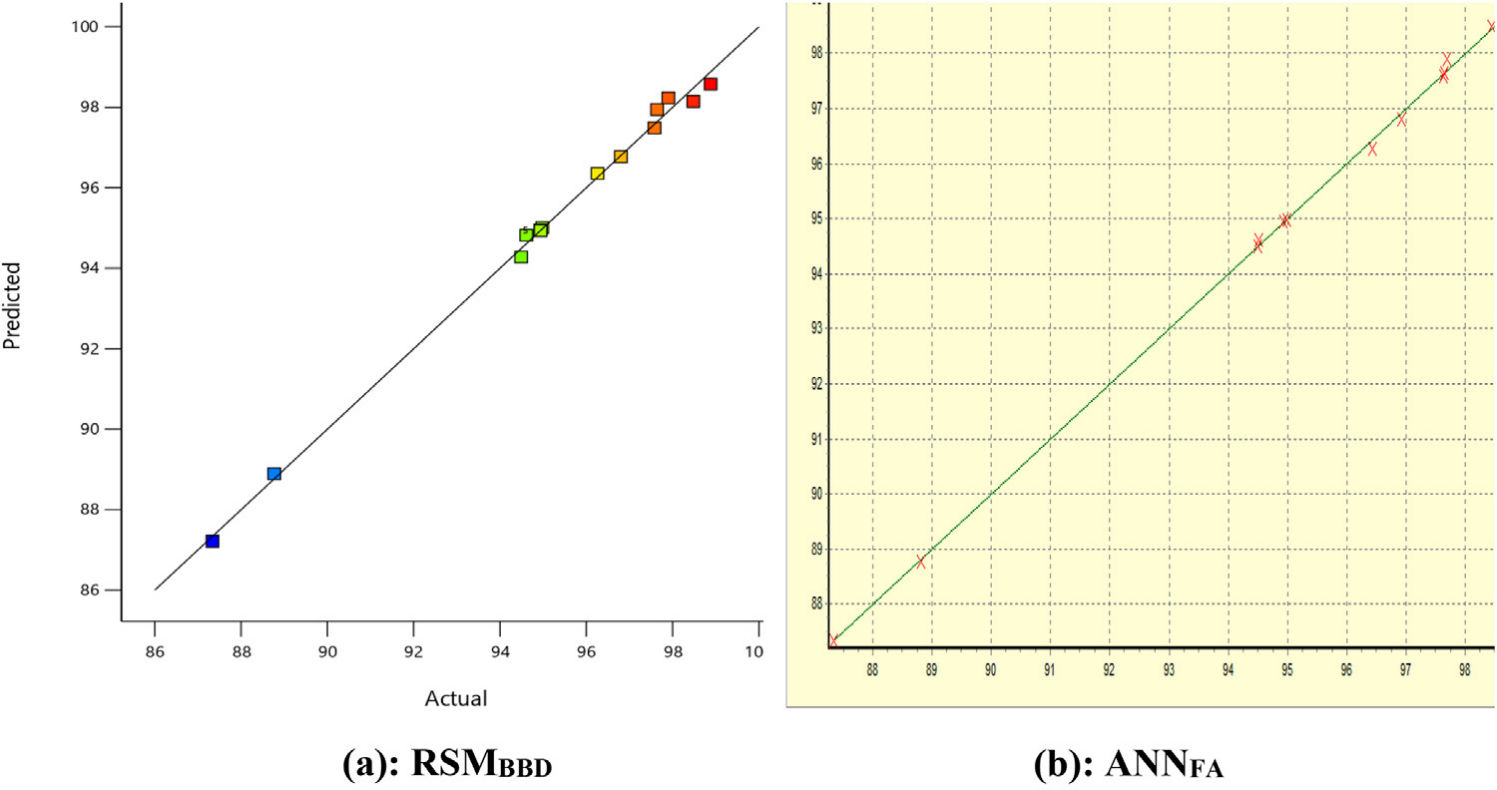
(a, b): The experimental vs. the predicted values.

**Fig. 8:**
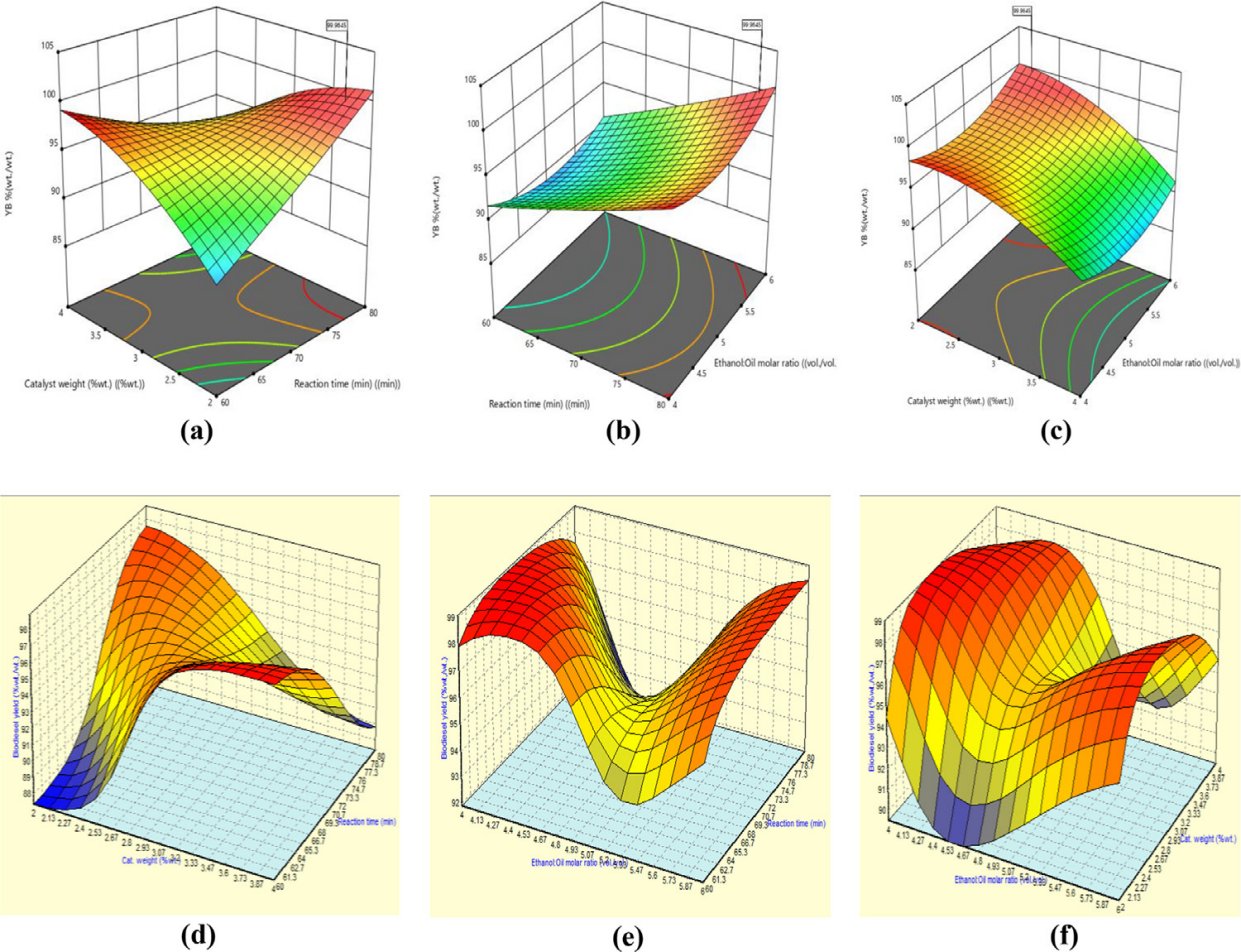
(a–c): RSMBBD three dimensional plots (d–f): ANNFA three dimensional plots.

The experimental data along with the predicted values by RSM_BBD_ and ANN_FA_ are presented in Fig. 7(a, b) while the ANOVA and the Fits statistical data are displayed in Table 7. The relationship between the interactive factors and the response (biodiesel yield) are presented inform of the three dimensional contour plots in Fig. 8[a–c] for RSM_BBD_ & Fig. 8[d–f] for ANN_FA_.

**Table 7:** Analysis of variance for regression analysis.

Source	Sum of squares	Df	Mean square	*F*-value	*p*-value	Level of significance
**Model**	150.71	9	16.75	214.16	<0.0001	significant
*X*_1_	0.5460	1	0.5460	6.98	0.0333	
*X*_2_	2.66	1	2.66	33.98	0.0006	
*X*_3_	0.7442	1	0.7442	9.52	0.0177	
*X*_1_×_2_	104.14	1	104.14	1331.92	<0.0001	
*X*_1_×_3_	1.61	1	1.61	20.63	0.0027	
*X*_2_×_3_	1.80	1	1.80	22.96	0.0020	
*X*_1_²	0.3213	1	0.3213	4.11	0.0823	
*X*_2_²	17.85	1	17.85	228.24	<0.0001	
*X*_3_²	23.03	1	23.03	294.55	<0.0001	
**Residual**	0.5473	7	0.0782	–	–	
Lack of fit	0.5473	3	0.1824	–	–	not significant
Pure error	0.0000	4	0.0000	–	–	
Cor. total	884.02	16	–	–	–	

**RSM_BBD_**			**ANN_FA_**			

*R*^2^	99.64%	–	*R*^2^	99.99%		
Adj. *R*^2^	99.17%	–	Adj. *R*^2^	99.99%		
Pred. *R*^2^	94.21%	–	Pred. *R*^2^	98.97%		
Std. Dev.	0.2796	–	Std. Dev.	2.071

The polynomial modeling equation for biodiesel prediction yield is established in Eq. (2).

Transfer model equation in terms of actual:

(2) $$\begin{array}{ccc} \text{BY}\%(\text{wt}./\text{wt}.) & = & 51.41+0.85X_{1}+52.00X_{2}-26.13X_{3}-0.51X_{1}X_{2}+0.06X_{1}X_{3}\\ & & -\;0.67X_{2}X_{3}+0.003X_{1}^{2}-2.06X_{2}^{2}+2.34X_{3}^{2} \end{array}$$

**Fig. 9:**
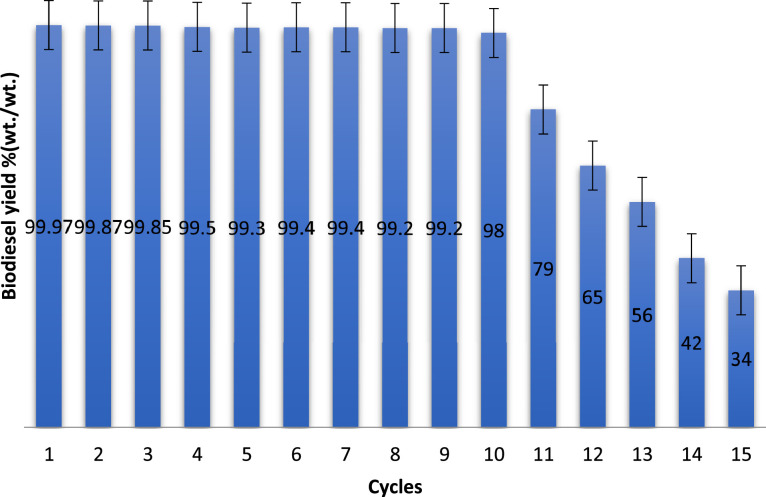
Catalyst reusability test plots.

The optimum predicted responses by RSM and ANN were validated in triplicate, and an average values of 99.02% (wt./wt.) [*X*_1_ = 78.42 (min), *X*_2_ = 2.19 (%wt.), and *X*_3_ = 5.969] and 99.97% (wt./wt.) [*X*_1_ = 70.41 (min), *X*_2_ = 5.40 (%wt.), and *X*_3_ = 6.00] were established. ANN in suit forced approach shows supremacy over the RSM in suit Box Behnken Design optimum validated yield. Furthermore, the regression parameters exhibited by ANN are better than that exhibited by RSM. Table 8 however accounted for the nature of biodiesel produced as compared with biodiesel recommended standard. Displayed in Fig. 9 are the data obtained during catalyst reusability tests.

**Table 8:** Qualities of the biodiesel.

Parameter	Biodiesel	[1]	[2]
Color@ 27 °C	Dark-brownish	–	–
Density (kg/m^3^) @ 27 °C	868	–	860–900
Viscosity @ 40 °C/ (mm^2^/s)	2.12	1.9–6.0	3.5–5.0
Moisture content (%)	<0.001	<0.03	0.02
%FFA (as oleic acid)	0.21	0.40 max	0.25 max
Acid value (mg KOH/g oil)	0.42	0.80 max	0.5 max
Iodine value (g I_2_/100 g oil)	72.50	–	120 max
Saponification value (mg KOH/g oil)	132.40	–	–
Peroxide value (meq O_2_/kg oil)	6.54	–	12.85
HHV (MJ/kg)	42.91	–	–
Cetane number	71.21	57 min	51 min

## 4 Experimental, Design, Materials and Methods

### 4.1 Materials

Freshly harvested pink solo pawpaw seed (*Carica papaya* seed) was collected from fruits sellers in Ughelli market, Warri, (Longitude: 5.324928° N, Latitude: 6.131293° E), Delta State, Nigeria. The eyes-like wet seeds were immediately oven dried at 240 °C for 2 h, milled with laboratory blender to smaller particle sizes (0.30 µm mesh size). The powder sample was then extracted with *n*-hexane in twenty (20) experimental runs, and the extracted oil was kept for further analysis.

10 kg of sweet corn pod was collected from maize seller in Ozoro (Longitude: 8.8403° N, Latitude: 7.0539° E), Delta State, Nigeria. The pod was washed with distilled water twice, and then sundried for 24 h to eliminate wetness, the wet-free pods were then oven dried at 150 °C for 8 h, and the dried pod was burnt in an open air. The burnt pod was then milled into powder using manual pounder; the milled pod powder was sieved into small particle size (0.25 µm mesh size). This was stored in a tight covered container for further processing. All chemical used were of standards grades with purities (ethanol: 99.5%; methanol: 99.85%; NaOH: 80%; HCl: 37%, sg: 1.18 g/mL; H_2_SO_4_: 98%, sg: 1.8 g/mL; KI: 69% with a 76% grade; *n*-hexane: 99.5%).

### 4.2 Methods

#### 4.2.1 Pink solo *Carica papaya* seed oil extraction

Pink solo *Carica papaya* oil (PSPO) was obtained using solvent extraction method with *n-* hexane as solvent due to its attribute such as simple recovery, non polar nature, low latent heat of vaporization, and high selectivity.

The extractor, 250 mL capacity was filled with *n*-hexane (150, 175, and 200 mL), the seed powder weight (25, 30, and 35 g) was measured with muslin bag placed in extraction chamber, and the extraction temperature was set at boiling point of *n*-hexane (156 °F). The reaction time was varied from one extraction time to another within the range of 50 to 60 min until the oil was completely extracted from the powder [3]. The extract was made free of excess *n*-hexane using rotavp, and the pure oil yield along with n-hexane recovered was computed. The recovery *n*-hexane was reused [4], and the pure oil was kept in a tight container for further process. The design of experiment was carried out by RSM, while the modeling and optimization of oil extraction and biodiesel synthesis were conducted using both ANN in suit forced approach and the RSM in suit BOX Behnken Design.

#### 4.2.2 Designs by RSM and ANN

Three factors were considered for oil extraction design namely: *Carica papaya* seed weight (CPSW), extraction time (ET), and the volume of solvent (VS). The design level, factors and their respective symbols were generated in 17 experimental runs [5], and were experimentally carried out. Variables optimization, transformation, process modeling, parameter evaluation, and optimum prediction of response was carried out step by steps through the test of significant, regression analysis, significant of probability test value (*P*-value <0.05), *F*-distribution (*f*-test), variance inflation factor (VIF), and the degree of freedom (df) [6]. The adequacy of the model was confirmed via regression parameters (*R*^2^, adj. *R^2^*, pred. *R^2^*, and Adeq. Precision). To examine the validity of the results, the experimental results and the predicted response were elucidated using predicted against actual plot. The relationship between the variables interactions and the response were examined through the 3-dimensional contour plots. These were also performed for ANN [7].

#### 4.2.3 Process modeling and optimization

To ascertain the effects of variable factors on the yield of oil, determine the linear regression parameters, the test of significant for good model fitness, and the effects of factors on the yield, was carried out via both Box–Behnken through RSM and force algorithm through ANN [[8], [9], [10]]. The optimum oil yield was validated at different variable conditions, and the result was compared and contrast. The mathematical model equation that explained the polynomial quadratic model between the responses and the variable factors were also presented (Eq. (3)).

(3) $$\begin{array}{ccc} OY\;\%\left(\text{wt}./\text{wt}.\right) & = & I+\alpha_{1}A+\alpha_{2}B+\alpha_{3}C+\alpha_{1}\alpha_{2}\text{AB}+\alpha_{1}\alpha_{3}\text{AC}+\alpha_{2}\alpha_{3}\text{BC}\\ & & +\;\alpha_{1}A^{2}+\alpha_{2}B^{2}+\alpha_{3}C^{2} \end{array}$$

where: I is the intercept, A,B,andCare the linear terms, AB,AC,andBCare the interaction terms, while the $A^{2},\;B^{2},\;\text{and}\;C^{2}\;$are the quadratic model terms.

The accuracy of the data by RSM was compared with ANN optimizer.

#### 4.2.4 Properties of pink solo *Carica papaya* oil (PSCPO)

The quality of the oil extracted was ascertained by determining the properties of the oil via AOAC, 1997 procedures [[11], [12]]. The fatty acid of the oil were also examined and recorded with a view to determine its suitability for biodiesel production.

#### 4.2.5 Acidified sweet corn stock powder catalyst characterization

The powder catalyst immersed in 0.1 HCl was characterized in other to ascertain the nature and quality of the catalyst. Also, the morphological structures and sizes, the acidic-base compound present; the functional groups associated with the catalysts on the transmittance, and the isotherm adsorptions were established. The morphological surface topography was study by SEM-EDX (model 6300F by JEOL solution for innovation located in USA), while the XRF-FT (model NEX CG, made by Rugaku, Austin, located in USA) was employed to confirm the hetero-compound present in the catalysts [13]. The FTIR with model 3,116,465, made in Japan was used to examine the functional groups and characteristic absorption bands. The ZETA potential analysis was carried out using Microtrac instruments that operate on the basis of Dynamic Light Scattering (DLS) to determine the surface charge of the particles [14]. TGA was used to determine the effect of thermal treatment on the acidified sweet corn stock powder based on weight loss, while XRD was used to ascertain the compounds present associated with acidic-alkaline [15].

#### 4.2.6 Synthesis of biodiesel

The oil was preheated at temperature of 80 °C for 60 min in a biodiesel reactor, acidified catalyst was weighed and added to a know volume of ethanol, and the mixture was stirred to achieved ethanol-catalyst mixture [[16], [17]]. The mixed ethanol-catalyst was added to preheated oil in the reactor, and the reaction was monitored at 70 °C for a known time. At the reaction completion, the product was allowed to stand in a separating funnel for 48 h to allow the gravity settling. At the end of 48 h, three layers were observed: the ethanol thin layer, the biodiesel thick layer, and the glycerol-catalyst layer [[18], [19]]. The layer was fractionally separated by removing glycerol-catalyst layers from the bottom of separating funnel, and the ethanol-biodiesel layers were washed with distilled water, dried over magnesium sulfate, and then filtered to obtained pure diesel. The pure biodiesel was obtained by computation using Eq. (4), while the catalyst was recycled from the glycerol, refined, centrifuge and reused [18]. The pure dried biodiesel was kept in a cleaned 5 L container for further analysis.

(4) $$Y\;\%\left(\text{wt}./\text{wt}.\right)=\frac{MW_{B}}{MW_{PSCPO}}\times 100$$

where;Y biodiesel yield, $MW_{B}$ molar weight of the produced biodiesel, $MW_{PSCPO}$ molar weight of the pink solo C*arica papaya* oil.

#### 4.2.7 Modeling and optimization of biodiesel production

In the production of biodiesel, three variables were considered (reaction time, catalyst weight, and ethanol/oil molar ratio) for the single stage process design using response surface methodology (RSM). The design considered generated a total of 17 experimental runs using Box–Behnken Design and was carried out in triplicate to obtained average experimental biodiesel yield [20]. The variables effects on biodiesel yield, linear regression parameters, the test of significant for good model fitness and the effects of factors on the yield were carried out via both Box–Behnken Design induced RSM and force algorithm induced ANN. The optimum biodiesel yield was validated at different variable conditions, and the result was compared and contrast [19]. The mathematical model equation that explained the polynomial quadratic model between the responses and the variable factors were also presented Eq. (5).

(5) $$YB\;\%\left(\text{wt}.\;/\;\text{wt}.\right)=\cap_{0}+\sum_{i=1}^{k}\cap_{i}X_{i}+\sum_{i=1}^{k}\cap_{ijz}X_{i}^{2}+\sum_{i=1}^{k}\cap_{ijz}X_{i}^{3}+\sum_{i<j}^{k}\cap_{ij}X_{i}X_{j}X_{z}+\epsilon$$

where: YB is the response, $\cap_{0}$ is the intercept, $\cap_{i}\mu_{i}$ is the linear coefficient, $\cap_{ijz}\mu_{i}^{2}$ is the quadratic terms, $\cap_{ijz}\mu_{i}^{3}$ the cubic terms, $\sum_{i<j}^{k}\cap_{ij}\mu_{i}\mu_{j}\mu_{z}$ the linear interactions, and ϵ is the residual error [5].

#### 4.2.8 Catalyst recycled, refining and reusability test

Catalyst was recycled, refine and reused for the synthesis of biodiesel. The reusability test was carried out in various cycles, a total of 15 cycles of reusability was performed, and the data are presented with errors bar using Microsoft office excel 2010 .lnk plots [20]. Initially, no significant reduction was notice until cycle 11 where a significant change was observed. Hence, catalyst reusability was altered. The data indicated that the catalyst is naturally stable and active and could serve as raw materials in petroleum and other oil industries.

## Limitations

The article is limited to the use of one variety of *Carica papaya* seed and one variety of maize stalks. The use of trial version of the design expert is also a limitation in this study.

## Ethics Statements

Not applicable. This work did not involve any types of animal or human studies.

## Declaration of Generative AI and AI-Assisted Technologies in the Writing Process

During the preparation of this work the author(s) used extractor, reactor, and magnetic stirrer in order to produce biodiesel. After using this tool, the author(s) reviewed and edited the content as needed and take(s) full responsibility for the content of the publication.

## Funding

This article receive no fund whatsoever from private or government organization.

## CRediT authorship contribution statement

**O. Nyorere:** Conceptualization, Methodology, Software, Writing – original draft. **S.I. Oluka:** Supervision, Data curation. **S.E. Onoji:** Supervision, Visualization, Investigation. **R. Nwadiolu:** Software, Validation, Writing – original draft. **T.F. Adepoju:** Writing – original draft.

## Data Availability

Dataset on Acido-biocatalysis of Agro Solid Wastes in Acidic Medium for the Conversion of Pink Solo Carica papaya Seed oil to Biodiesel Fuel (Original data) (Mendeley Data).
